# The pattern of recurrence of adenocarcinoma of the oesophago-gastric junction

**DOI:** 10.1038/sj.bjc.6600252

**Published:** 2002-04-22

**Authors:** J Wayman, M K Bennett, S A Raimes, S M Griffin

**Affiliations:** The Northern Oesophago-Gastric Cancer Unit, University of Newcastle upon Tyne, The Royal Victoria Infirmary, Newcastle upon Tyne NE1 4LP, UK

**Keywords:** gastric carcinoma, oesophageal carcinoma, surgical treatment, recurrence

## Abstract

Knowledge of the pattern of recurrence of surgically treated cases of adenocarcinoma of the oesophago-gastric junction is important both for better understanding of their biological nature and for future strategic planning of therapy. The aim of this study is to demonstrate and compare the pattern of dissemination and recurrence in patients with Type I and Type II adenocarcinoma of oesophago-gastric junction. A prospective audit of the clinico-pathological features of patients who had undergone surgery with curative intent for adenocarcinoma of oesophago-gastric junction between 1991 and 1996 was undertaken. Patients were followed up by regular clinical examination. Clinical evaluation was supported by ultrasound, computerised tomography, radio-isotope bone scan, endoscopy and laparotomy each with biopsy and histology where appropriate. One hundred and sixty-nine patients with oesophago-gastric junction tumours (94 Type I and 75 Type II) have been followed up for a median of 75.3 (57–133) months. One hundred and three patients developed proven recurrent disease. The median time to recurrence was 23.3 (14.2–32.4) months for Type I and 20.5 (11.6–29.4) for Type II cancers. The most frequent type of recurrence was haematogenous (56% of Type I recurrences and 54% of Type II) of which 56% were detected within 1 year of surgery. The most frequent sites were to liver (27%), bone (18%) brain (11%) and lung (11%). Local recurrence occurred in 33% of Type I cancer and 29% of Type II recurrences. Nodal recurrence occurred in 18 and 25% of Type I and Type II cancer recurrences, most frequently to coeliac or porta hepatis nodes (64%). Only 7% of Type I and 15% of Type II cancer recurrences were by peritoneal dissemination. Type I and Type II adenocarcinoma of the oesophago-gastric junction have a predominantly early, haematogenous pattern of recurrence. There is a need to better identify the group of patients with small metastases at the time of diagnosis who are destined to develop recurrent disease in order that they may be spared surgery and those with micro metastases in order that they can be offered multi-modality therapy including early post operative or neo-adjuvant chemotherapy.

*British Journal of Cancer* (2002) **86**, 1223–1229. DOI: 10.1038/sj/bjc/6600252 www.bjcancer.com

© 2002 Cancer Research UK

## 

Cancer registry and hospital data shows that there has been a change in the topography of gastric cancer and topography and morphology of oesophageal cancer with an increasing proportion of cases of adenocarcinoma of the gastric cardia and lower oesophagus i.e. adenocarcinoma of oesophago-gastric junction (Antonioli and Goldman, 1982; Meyers *et al*, 1987; Hesketh *et al*, 1989; Powell and McConkey, 1990; Blot *et al*, 1991; Pera *et al*, 1993; McKinney *et al*, 1995). Similarities in terms of histopathology and epidemiological trends have been identified (Kalish *et al*, 1984; Wang *et al*, 1986; MacDonald and MacDonald, 1987; Powell and McConkey, 1992; Heidl *et al*, 1993; Siewert and Stein, 1998) and have led some observers to consider the two cancers as one disease with similar aetiologies (Clark *et al*, 1994).

A consensus conference of the International Gastric Cancer Congress declared that cancers arising at or close to the oesophago-gastric junction should be considered separately from gastric and other oesophageal cancers and a new classification system for junctional cancers was devised (Table 1

**Table 1:**
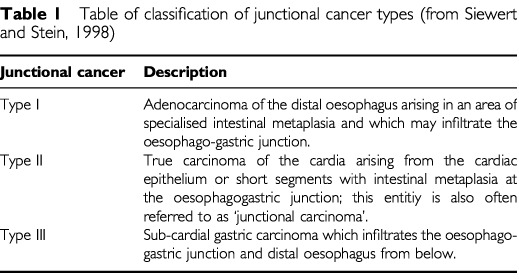
Table of classification of junctional cancer types (from Siewert and Stein, 1998)

). According to this classification, Type I and II junctional cancers are becoming the predominant oesophago-gastric cancer type. While the pattern of recurrence following resection of oesophageal squamous cancer and distal gastric cancer has been well documented (Gunderson and Sosin, 1982; Law *et al*, 1996) the natural history of adenocarcinoma of the oesophago-gastric junction is yet to be defined. Accurate knowledge of the temporal, anatomical and biological pattern of recurrence following surgery has important implications for planning therapeutic strategies. The purpose of this study is to analyse the pattern of recurrence in patients who have undergone curative surgical resection for Type I and Type II adenocarcinoma of the oesophago-gastric junction.

## MATERIALS AND METHODS

A prospective audit was performed of all patients undergoing surgery with curative intent for Type I and Type II adenocarcinoma of the oesophago-gastric junction. Between 1991 and 1996, 610 patients were referred and assessed. Of these 378 (62%) were selected for surgery with the intention of curative resection. Overall, this group consisted of 195 patients with oesophageal cancer and 183 patients with gastric cancer. Among these, there was a total of 94 cases of adenocarcinoma of the lowest 5 cm of the oesophagus (Type I junctional cancers) and 75 of the gastric cardia (Type II junctional cancers) who underwent potentially curative resection. All patients underwent detailed pre-operative assessment prior to selection for surgery. This assessment included appraisal of tumour stage and resectability by chest X-ray, endoscopy, abdominal ultrasound scan and thoraco-abdominal spiral CT scan. Tumour site was determined at endoscopy along with a record of proximal and distal margins and the position of the oesophagogastric junction. Clinical and pathological details, based on a standardised reporting protocol were recorded

The operative approach and extent of procedure performed for tumours around the oesophago-gastric junction was tailored to each case with respect to tumour stage and patient fitness. Ninety two patients with Type I junctional cancers underwent two stage sub-total oesophagectomy with two field lymphadenectomy (Griffin, 1998) and two underwent a thoraco-abdominal gastro-oesophagectomy with D2 lymphadenectomy (Heitmiller, 1992). Of the Type II junctional cancer patients, one underwent a two stage oesophago-gastrectomy and 74 underwent a total gastrectomy with Roux-en-Y oesophago-jejunal anastomosis: 18 had a D1 and 56 had D2 lymphadenectomy. Additionally seven patients had splenectomy; two along with distal pancreatectomy. Strict adherence to the Japanese Rules for Gastric Cancer Surgery recommended by the Japanese Research Society for Gastric Cancer (JRSGC) was observed for description of lymphadenectomy. Only if all N1 and N2 Stations were resected *en bloc* with the stomach, was the resection deemed D2. For those who fell short of this, most commonly for failure to remove splenic hilar nodes, D1 was used. In effect, our ‘D1’ resections were invariably more extensive than simply the N1 *en bloc* resection as described by the JRSGC. The approach used was typically the thoraco-abdominal approach in the former part of the series and latterly changed to a transhiatal approach with an associated reduction in peri-operative complications (Wayman *et al*, 1999). Neo-adjuvant or adjuvant therapy was not used in any of this group of patients.

All patients underwent clinical review at 6 weeks following discharge and 3 monthly thereafter. Clinical review consisted of history and abdominal examination. No investigations were routinely instituted as long as the patient remained symptomatically well with no abnormality on clinical examination. A protocol for investigation of symptomatic recurrence was developed. In this, primary symptoms prompted investigation as shown in Table 2

**Table 2:**
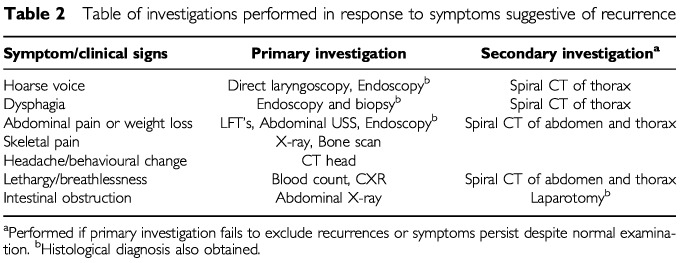
Table of investigations performed in response to symptoms suggestive of recurrence

. In addition, autopsy information was available in eight patients, of whom six had recurrent disease. Post mortem examination was not routinely requested in patients with recurrent disease detected prior to death.

All data were compiled prospectively onto proformas and transferred onto a database (Paradox 3.1 for DOS). Disease free survival was calculated by the method described by Kaplan–Meier with univariate comparisons in median survival by the log rank test and multivariate analyses by Cox Regression. Comparison of continuous data between groups was by the Mann–Whitney *U*-test and ordinal data by the χ^2^ test. Analyses were performed using the Statistics Package for the Social Sciences (SPSS 6.1.3 for Windows®, Chicago, IL, USA).

## RESULTS

One hundred and sixty-nine patients with tumours of the gastro-oesophageal junction who underwent resection with curative intent were followed and studied (Table 3

**Table 3:**
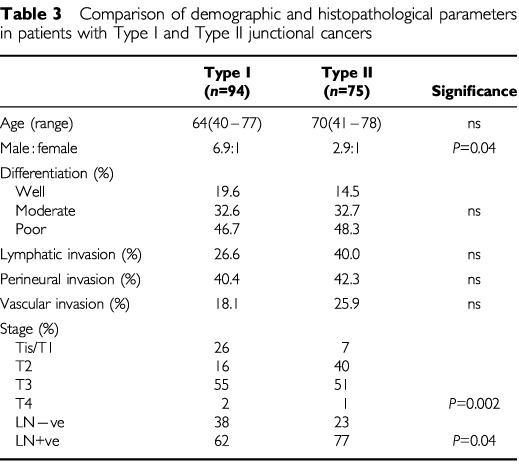
Comparison of demographic and histopathological parameters in patients with Type I and Type II junctional cancers

). There was a higher male to female ratio in patients with Type I junctional cancer. There was a higher proportion of tumours confined to the mucosa and sub-mucosa in Type I cancers compared to Type II (26% *vs* 7%; χ^2^=17.2, 4 *df*, *P*=0.002) and a higher proportion of lymph node negative patients (38% *vs* 23%; χ^2^= 4.0, 1 *df*, *P*=0.04). There was no significant difference in the other histopathological parameters measured. In the time period studied, the median yield of the two-field lymphadenectomy was 13 (4–33), with involvement of median 4 (1–20), the D1 lymphadenectomy yielded a median of 21(4–53) nodes with a median positivity of 6 (1–43) while those described as D2 resection yielded 21 (1–52) with a positivity of yield of 5 (1–30).

Median follow up of patients was 75.3 months (range 57–133). Of the 169 patients who underwent resection with curative intent, 103 patients developed recurrent disease; 55 out of 94 of Type I and 48 out of 75 Type II junctional tumours. A further 19 patients have died without evidence of recurrence including five within 30 days of surgery, and a further four within 3 months of surgery (Table 4

**Table 4:**
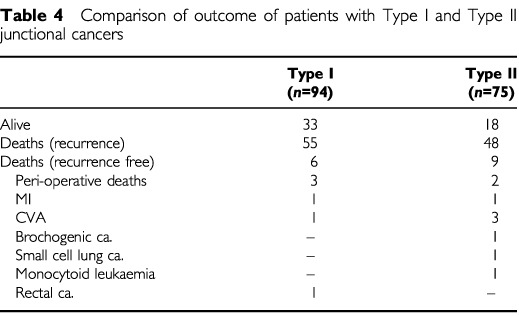
Comparison of outcome of patients with Type I and Type II junctional cancers

).

**Figure 1:**
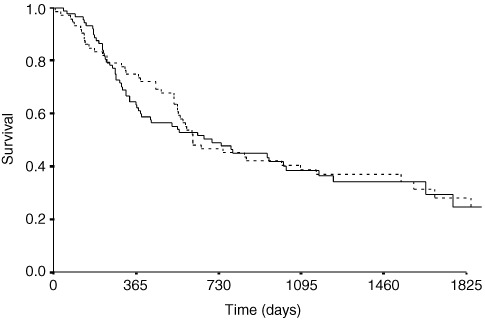
Kaplan–Meier plot of recurrence free survival of cases of Type I (broken line) and Type II (straight line) junctional cancers.

The median, recurrence free survival for Type I and Type II junctional cancers was 699 (425–973) days *vs* 615 (348–882) (χ^2^= 0.04, 1 df *P*=0.84) (Figure 1

**Figure 2:**
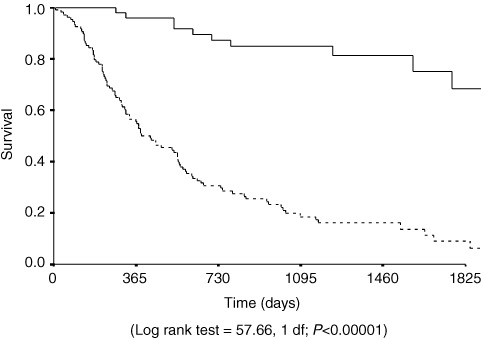
Kaplan–Meier plot of recurrence free survival of cases of Lymph node positive (broken line) and negative (straight line) junctional cancers.

). The median time to death following recurrence was 61 (0–782) days, but highly variable and was similar for Type I (46 (0–445)) and Type II (62 (0–782)) cancers. Time to death for each of the patterns of recurrence was not significantly different; haematogenous (52 (0–445)), lymphatic (143 (19–782)), local (63 (0–339)) and peritoneal spread (38 (14–184)) days. Lymph node status, depth of invasion, grade of differentiation and the presence of lymphatic, peri-neural or vascular invasion and the presence of positive resection margins were all associated with poorer disease free survival on univariate analysis (Table 5

**Table 5:**
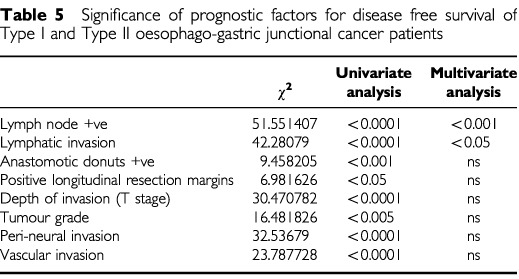
Significance of prognostic factors for disease free survival of Type I and Type II oesophago-gastric junctional cancer patients

). On multivariate analysis of disease free survival only lymph node status and histological evidence of lymphatic invasion were significant independent prognostic factors (Table 5, Figure 2). Extent of lymphadenectomy in the group of patients undergoing total gastrectomy (‘D1’ *vs* ‘D2’) was not significantly related to timing or pattern of recurrence. Sub-analysis of the lymph node positive group revealed nodal burden (where high burden was regarded as four or more positive) was a significant prognostic factor (Figure 3

**Figure 3:**
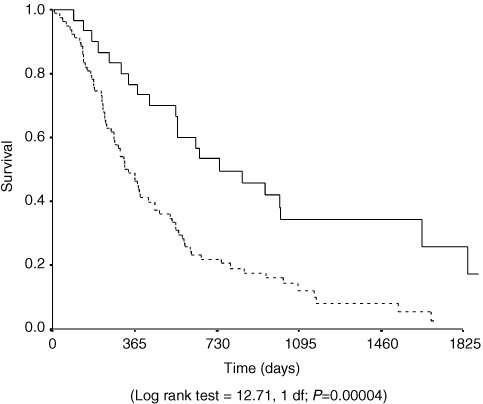
Kaplan–Meier plot of recurrence free survival of cases of Lymph node positive junctional cancers with low (<4 nodes positive) (straight line) and high nodal burden (broken line).

) (*P*=0.0004).

The distribution of recurrent disease was similar in Type I and Type II junctional cancers (Table 6

**Table 6:**
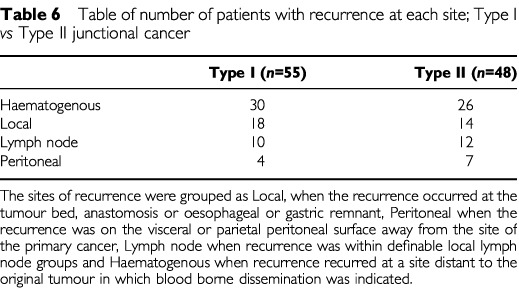
Table of number of patients with recurrence at each site; Type I *vs* Type II junctional cancer

). The commonest type of recurrence in both was haematogenous, occurring in 56% of Type I and 54% of Type II junctional cancer recurrences. Within the group of patients with haematogenous recurrence the most frequent metastatic sites were bone and hepatic (Table 7

**Table 7:**
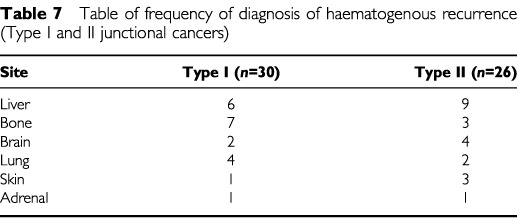
Table of frequency of diagnosis of haematogenous recurrence (Type I and II junctional cancers)

). Local recurrence occurred in 32 patients (33% of Type I recurrence cases, 29% of Type II recurrence cases) at the tumour bed including the mediastinum and oesophago-jejunal or oesophago-gastric anastomosis. In a further 22 cases, local lymph node groups could be distinguished on USS (*n*=7) or spiral CT scan (*n*=15). The most common lymph node groups involved were are listed below (Table 8

**Table 8:**
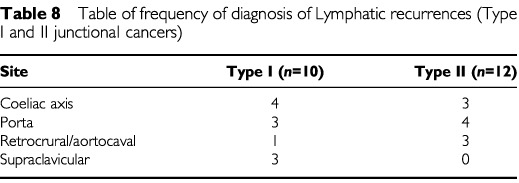
Table of frequency of diagnosis of Lymphatic recurrences (Type I and II junctional cancers)

). In addition, pulmonary lymphangitis carcinomatosis was a feature of recurrence in four patients. Distant peritoneal disease was diagnosed in 7% of Type I and 14.5% of Type II junctional cancer recurrences by a laparotomy (*n*=3), spiral CT scan (*n*=6) or autopsy (*n*=2).

Eleven patients (15%) had positive resection margins of which six cases had tumour within the anastomotic donuts. The presence of positive resection margins was associated with poorer disease free survival although this was dependent on lymph node status (Table 5). The site of recurrence in these patients was local in only four, haematogenous in seven (one case having both lymph node and haematogenous recurrence), peritoneal in two and lymphatic in one. Similarly, the presence of positive lymph nodes in the resected specimen was associated with haematogenous recurrence (*n*=45) more than lymphatic (*n*=16), local (*n*=20), and peritoneal (*n*=11) recurrence.

**Figure 4:**
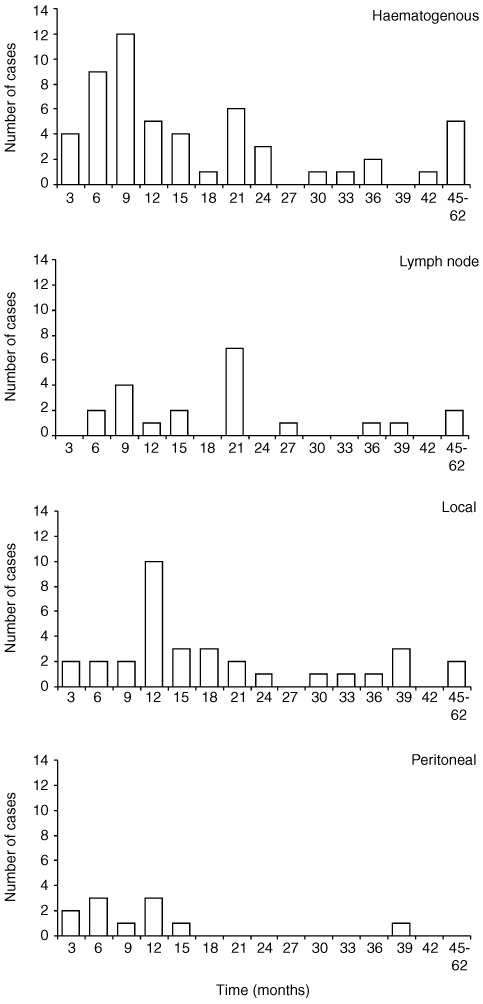
Bar charts illustrating timing of each mode of recurrence. Time in months on x axis. Number of cases with recurrence on y axis.

Haematogenous recurrence in particular tended to occur rapidly after surgery; median time to recurrence haematogenous (360 (10–1844)), lymphatic (547 (181–1162)), local (364 (146–1685)) and peritoneal spread (149 (132–1091)) days (Figure 4

). Patients in whom recurrent disease was detectable within the first year were considered separately since they arguably represent a group of outright treatment failures. This group of patients (*n*=50) showed a predominance of haematogenous recurrence (46%). Fifty-six per cent of haematogenous recurrence (30 out of 57) occurred within 12 months. Lymph node involvement was the only significant prognostic marker for first year recurrence of any site compared with later recurrence (*P*>0.0001) and within this group 77% had had a high nodal burden.

## DISCUSSION

Natural and surgically manipulated pathways of tumour spread determine subsequent patterns of recurrence. In oesophago-gastric cancer direct extension can lead to local recurrence, lymphatogenous spread to local lymph recurrence, haematogenous spread to recurrence at distant sites and transcoelomic spread to peritoneal recurrence. The results of this study suggest that Type I and Type II junctional cancers have a similar pattern of dissemination and recurrence which is different from that reported for distal gastric cancer and oesophageal squamous carcinoma.

Caution must be applied when comparing these findings with previously reported work. The criteria used for detecting and diagnosing recurrence in this clinically based study are inferior to studies based on a systematic planned ‘Second look’ laparotomy, symptomatic re-look laparotomy or autopsy. Only 6% of recurrences in this series were diagnosed by post mortem examination. This study is therefore biased towards diagnosing symptomatic recurrence and that which can be most easily imaged. It may particularly underestimate the incidence of small peritoneal and local recurrences.

The categorisation in this study into local, lymphatic, peritoneal and haematogenous recurrence is somewhat artificial. There is clearly likely to be considerable overlap between local and lymphatic recurrence due to the difficulty in distinguishing lymph node groups on CT scan. Also, some cases of local recurrence may represent locally confined peritoneal dissemination or a feature of more widespread peritoneal dissemination too subtle to be detected by CT and USS, both of which are known to be inaccurate in diagnosing peritoneal dissemination. Nevertheless, haematogenous dissemination is, in this study, more objectively defined.

The earliest evidence for the pattern of failure following gastric resections for cancer comes from autopsy studies. McNeer found that of 92 patients undergoing ‘curative sub-total gastrectomy’ 80.4% were found to have evidence of recurrence at post-mortem (McNeer *et al*, 1951). Among these the great majority were local recurrences; 65.2% had recurrence in the gastric remnant, gastroenterostomy or duodenum while 52.2% had recurrence in peri-gastric nodes and stomach bed, 30% had peritoneal seedlings and 40% had haematogenous recurrence. Such post mortem data lacks any information about timing of recurrence.

Wangensteen, with his policy of systematic planned re-operations following ‘curative’ operations was the first to be able to map the incidence and precise areas of failure of gastric cancer resections before they became clinically apparent (Wangensteen *et al*, 1954). Although such a policy proved to be of little therapeutic value, this and a subsequent similar analysis by Gunderson and Sosin (1982) provided invaluable information and moulded opinion about the nature and pattern of gastric cancer recurrence. In their work, they found that 87.8% of failures were in some part contributed to by local and/or regional recurrence defined as recurrence in local lymph node groups or tumour bed; 53.7% had peritoneal seedlings, 29.3% of which were diffuse. Of 105 patients followed up, only five had isolated distant metastases. At the time of this work, there was a general acceptance that the majority of patients with gastric cancer would die with locally recurrent disease and the purpose of such studies was to define the anatomy of this recurrence with a view to planning adjuvant radiotherapy schedules. More recent data for the pattern of failure of gastric cancer comes from Japan and Korea. There the disease is still predominantly of the distal stomach. In these series peritoneal recurrence remains the predominant cause of failure (Maehara *et al*, 2000; Yoo *et al*, 2000). Our results suggest that adenocarcinoma of the oesophago-gastric junction has a different natural history which should prompt a re-evaluation of adjuvant and neo-adjuvant therapies.

The pattern of recurrence of oesophageal cancer has been studied but has previously concentrated on squamous cell carcinoma (Law *et al*, 1996). Data from Hong Kong on 108 patients undergoing oesophagectomy and two-field lymphadenectomy with curative intent demonstrated recurrence in 56. Two thirds of patients with recurrence presented within the first year. The authors categorised recurrence as primarily intra- (equivalent to local recurrence in our study) or extra-thoracic. The majority of recurrences were extra-thoracic (44 *vs* 33 out of 56). Of the extrathoracic recurrences, 28 were by haematogenous dissemination to distant organs and 17 to lymph nodes; specifically cervical (*n*=12) and abdominal (*n*=5). The incidence of identified local recurrence (25%) is much greater than in our study for adenocarcinoma of the lower oesophagus (9%) or oesophago-gastric junction (15%). This may be partly to do with a higher proportion of early cancers in our Type I group. However, even as a proportion of those patients with recurrence, local recurrence is more common for squamous cancer (48%) than our finding with adenocarcinoma of the lower oesophagus (27%) or the gastric cardia (38%).

There may be many reasons for differences between the findings of this study compared to historical observations. The more radical nature of the surgery performed with perhaps greater attention to lymphadenectomy may account for the improved local disease control. The radical two-field lymphadenectomy appears to give good local disease control (Dresner and Griffin, 2001) and the total gastrectomy with D1 or D2 lymphadenectomy practised for the junctional cancers may also confer better local disease control than the more traditional distal and subtotal gastric resections. There are some conflicting reports suggesting that lymphadenectomy confers no survival benefit (Hulscher *et al*, 2001) however such studies are either too small with insufficient statistical power, not stratified by tumour stage or lack standardisation of technique. The choice of operation and extent of lymphadenectomy may have some influence on the pattern of recurrence. The majority of Type II cancers underwent total gastrectomy with abdominal lymphadenectomy and yet there are some who would suggest that mediastinal lymphadenectomy is appropriate in such cases. Our series did not have any examples of mediastinal recurrence. The other problem with total gastrectomy is the difficulty with proximal tumour clearance. In our series, following total gastrectomy, incomplete resection margin occurred in 15% of cases. In such cases, it may have been preferable to perform sub-total gastro-oesophagectomy. However this would compromise the abdominal lymphadenectomy. Interestingly, the pattern of recurrence in such patients was that which one would anticipate from the lymph node status. All had heavy nodal burden and resection was ultimately palliative with early systemic, not local recurrence. One might argue that for such patients, destined to have early recurrence, sparing them thoracotomy may have ultimately been to their benefit rather than their detriment.

It may also be that lymphadenectomy, if it does not confer survival benefit, may still alter the pattern of recurrence and be the explanation for the relatively lower incidence of local recurrence compared with historical data. The high incidence of early, haematogenous recurrence suggests genuine biological differences between the now predominating adenocarcinoma of the oesophago-gastric junction and the previously described squamous carcinoma of the oesophagus and mainly distal gastric cancers. It is somewhat difficult to draw contemporary comparisons as the incidence of more distal gastric cancer has fallen significantly in the West in the last two decades. Data from Japan, where radical lymphadenectomy has been common place for longer, would suggest that the predominant site of recurrence in distal gastric cancers remains local despite lymphadenectomy.

There was a significant difference in the stage at diagnosis of Type I and Type II junctional cancers in this study. The relatively higher incidence of lymph node negative and T1 cancers in the Type I group may be explained by their earlier presentation through Barrett's surveillance programmes at this unit and other tertiary referrals. Otherwise the similarity in their natural history, age distribution and histological characteristics suggest that Type I and Type II junctional cancers are biologically similar tumours. Other groups have identified pathological and demographic similarities between these cancers which contrast with differences between cardia and more distal stomach cancers (Kalish *et al*, 1984; Wang *et al*, 1986; MacDonald and MacDonald, 1987). Interestingly, our results do suggest differences with respect to the sex ratio. This may be explained by contamination of the Type II junctional group with Type III proximal third gastric cancers inherent in the retrospective reclassification of tumour type.

During our study period, the system of classification according to the IGCA was not available. Our study reclassifies, in retrospect, tumours previously classified according to ICD 9 codes as adenocarcinoma of the lower oesophagus (ICD 1505) and adenocarcinoma of the oesophagogastric junction/gastric cardia (ICD 1510). There is a risk of confusion in the data by such reclassification. Indeed this may also be a consideration of prospective classification. In our series we have two levels of control to minimise such cross-classification. The first is that in addition to site codes, according to the standardised staging protocol used, measurements were made at the time of endoscopy which can be used to accurately classify tumour position. The position of the diaphragm and whether the tumour was visible at the hiatus on retroflexion of the endoscope were also recorded. In addition, accurate position of the tumour was confirmed by the pathologist who also followed a standardised protocol for dissection and reporting of cases.

The early, haematogenous pattern of recurrence identified in this study for adenocarcinoma of the oesophago-gastric junction has important clinical implications. There are two groups of patients whom we must try to identify. There are those who already have metastatic disease at the time of diagnosis but who are not being identified by current routine staging techniques and those who have micrometastases. The former group are unlikely to be cured and may die early without benefiting from radical treatment. In such cases consideration should be given to non-operative treatment. For the latter group if they are elderly or unfit, non-operative treatment may be considered while for those in whom surgery is appropriate, treatment strategies designed to combat the early systemic recurrence should be considered. In such patients, inclusion in trials of systemic, neo-adjuvant therapy should be considered. Undoubtedly, chemotherapy will have an important role in any such therapeutic regime in order to control the early, haematogenous phase of recurrent disease.

Staging protocols should be reviewed. We are currently piloting, with encouraging results, a policy of radio-isotope bone scans before undertaking radical treatment. O'Sullivan *et al* (1999) have demonstrated that micrometastases are detectable in bone marrow in as many as 88% of cases of even early disease. There is no evidence that these findings correlate with subsequent haematogenous recurrence nor that their prognostic significance is independent of lymph node status. For these reasons, we do not use the technique in clinical practice. The presence of lymph node spread at the time of surgery was the strongest and only independent predictor of recurrence and specifically early haematogenous recurrence in our series. Our results have shown that pathologically assessed high lymph node burden is associated with the highest incidence of early haematogenous recurrence. Identification of high nodal burden pre-operatively may be a means of identifying those patients with micrometastases at risk of early recurrence. We now pay close attention to nodal burden as assessed pre-operatively by endoscopic ultrasound scan and CT.

In conclusion, the pattern of recurrence of Type I and Type II adenocarcinoma of the oesophagogastric junction is similar and characterised by early systemic recurrence especially in patients with lymph node disease at the time of surgery. Further research is required to better identify those with metastatic disease at the time of diagnosis who will not benefit from surgery and those most likely to have micrometastatic disease who may benefit from systemic neo-adjuvant chemotherapy as part of the multimodality treatment of this cancer.
